# The effect of sleep apnea severity on cardiac autonomic activity during night time in obstructive sleep apnea patients: O efeito da gravidade da apneia do sono sobre atividade cardíaca autonômica durante o período noturno em pacientes com apneia obstrutiva do sono

**DOI:** 10.1590/1516-3180.2015.0189070116

**Published:** 2016-06-27

**Authors:** Gulay Ozkececi, Sevinc Sarinc Ulasli, Onder Akci, Alaettin Avsar, Mehmet Unlu, Ersel Onrat

**Affiliations:** I MD. Assistant Professor, Department of Cardiology, Faculty of Medicine, Afyon Kocatepe University, Afyonkarahisar, Turkey.; II MD. Associate Professor, Department of Pulmonary Diseases, Faculty of Medicine, Hacettepe University, Ankara, Turkey.; III MD. Professor, Department of Cardiology, Faculty of Medicine, Afyon Kocatepe University, Afyonkarahisar, Turkey.; IV MD. Professor, Department of Pulmonary Diseases, Faculty of Medicine, Afyon Kocatepe University, Afyonkarahisar, Turkey.

**Keywords:** Heart rate, Arrhythmias, cardiac, Death, sudden, cardiac, Sleep apnea syndromes, Sleep apnea, obstructive, Frequência cardíaca, Arritmias, cardíacas, Morte súbita cardíaca, Síndromes da apneia do sono, Apneia do sono tipo obstrutiva

## Abstract

**CONTEXT AND OBJECTIVE:**

Impaired autonomic cardiac function is an important consequence of obstructive sleep apnea (OSA). This impairment is mainly due to intermittent hypoxia episodes following apneas. However, the impact of apnea severity on autonomic cardiac function remains unclear. The aim of this study was to evaluate the relationship between the severity of sleep apnea and heart rate turbulence (HRT) and heart rate variability (HRV) in OSA.

**DESIGN AND SETTING:**

Observational cross-sectional study conducted in the Departments of Cardiology and Pulmonary Diseases, Afyon Kocatepe University, Turkey.

**METHODS:**

106 patients with OSA and 27 healthy volunteers were enrolled. Based on apnea hypopnea index (AHI) values, obstructive sleep apnea severity was classified as follows: mild OSA (AHI ≥ 5 and < 15), moderate OSA (AHI ≥ 15 and ≤ 30) and severe OSA (AHI > 30). HRV and HRT parameters were assessed via 24-hour digital Holter electrocardiogram recordings for all subjects.

**RESULTS:**

HRV and HRT results were significantly lower among OSA patients than among control subjects (P < 0.05). However, there were no significant differences in HRT and HRV between the three patient subgroups. Correlations did emerge between AHI and the NN-interval parameter RMSSD and between oxygen desaturation and turbulence slope (respectively: r = -0.22, P = 0.037; and r = -0.28, P = 0.025).

**CONCLUSION:**

HRT and HRV results deteriorate in OSA. Correlations between apnea severity and these parameters seem to be present.

## RESUMO

**CONTEXTO E OBJETIVO:**

Função autonômica cardíaca prejudicada é consequência importante da apneia obstrutiva do sono (AOS). Este prejuízo deve-se principalmente a episódios de hipóxia intermitente após apneias. No entanto, o impacto da gravidade da apneia na função cardíaca autonômica permanece obscuro. O objetivo deste estudo foi avaliar a relação entre gravidade da apneia do sono com turbulência da frequência cardíaca (TFC) e variabilidade da frequência cardíaca (VFC) em pacientes com AOS.

**DESENHO E LOCAL:**

Estudo observacional transversal conduzido nos Departamentos de Cardiologia e Doenças Pulmonares, Afyon Kocatepe University, Turkey.

**MÉTODOS:**

106 pacientes com AOS e 27 voluntários saudáveis foram recrutados. Com base nos valores do índice de apneia-hypopneia (IAH), a gravidade da apneia obstrutiva do sono foi classificada assim: AOS leve (IAH ≥ 5 e < 15), AOS moderada (IAH ≥ 15 e ≤ 30) e AOS grave (IAH > 30). Parâmetros da VFC e TFC foram avaliados por meio de gravações de eletrocardiograma digital Holter de 24 horas para todos os sujeitos.

**RESULTADOS:**

Os resultados da VFC e TFC foram significativamente menores nos pacientes com OSA, em comparação com indivíduos controle (P < 0,05). No entanto, não houve diferenças significativas em VFC e TFC, entre os três subgrupos de pacientes. Correlações surgiram entre IAH e o parâmetro do intervalo-NN, RMSSD, e entre dessaturação de oxigênio e declive da turbulência (respectivamente; r = -0,22, P = 0,037; e r = -0,28, P = 0,025).

**CONCLUSÃO:**

Os resultados da VFC e TFC deterioram em AOS. Parece haver relação entre a gravidade da apneia e tais parâmetros.

## INTRODUCTION

Obstructive sleep apnea (OSA) is characterized by recurrent total apnea or partial hypopnea due to narrowed upper airways during sleep.^1^ The current estimates for the prevalence of moderate-to-severe sleep-disordered breathing (apnea-hypopnea index, AHI, measured as events/hour, ≥ 15) are 10% among 30-49 year-old men; 17% among 50-70 year-old men; 3% among 30-49 year-old women; and 9% among 50-70 year-old women, according to the study by Peppard et al.^2^ The prevalence of obstructive sleep apnea syndrome among Brazilian railroad workers has been found to be 35%.^3^

OSA is associated with cardiovascular diseases, including cardiac arrhythmias.^4^ myocardial infarction,^5^ chronic heart failure^6^ and pulmonary hypertension.^7^ The mechanism underlying cardiovascular diseases is complex and not fully understood in relation to OSA. Arrhythmias are considered to arise from changes in the cardiac autonomic balance due to hypoxia ­during apnea.^8^

There is a significant correlation between autonomic dysfunction and cardiovascular mortality.^9^ Baroreceptor reflex sensitivity, heart rate turbulence (HRT) and heart rate variability (HRV) are parameters reflecting cardiac autonomic functions. HRV and baroreceptor reflex sensitivity are believed to evaluate different aspects of autonomic control. While a moderate relationship has been determined between HRV and baroreceptor reflex sensitivity, there is a strong relationship between HRT and baroreceptor reflex sensitivity. Therefore, it has been suggested that HRT should be used as an evaluation parameter instead of baroreceptor reflex sensitivity.^10^^,^^11^

HRV^12^^,^^13^ and HRT^14^ provide important information regarding autonomic cardiac function. HRV measures the oscillation in successive cardiac cycles as well as the oscillations between instantaneous heart rates.^13^ Previous studies have determined that a reduction in HRV is predictive of increased cardiac mortality.^15^ HRT is defined as the sinus rhythm cycle-length fluctuation after isolated premature ventricular beats.^16^ HRT evaluation has been deemed appropriate for risk estimation after acute myocardial infarction^17^ and as a prognostic evaluator for heart failure^18^ and other pathological conditions.^16^

Autonomic cardiac functions in OSA patients have been the focus of attention for researchers. Previous studies revealed deteriorations in HRT and a relationship between HRT and the severity of the apnea.^19^^,^^20^^,^^21^ However, the results relating to HRV in these patients have been divergent; reductions and no changes in HRV have been reported in different studies.^20^^,^^21^^,^^22^

## OBJECTIVE

Therefore, the aims of the present study were to investigate whether both HRT and HRV parameters are impaired in patients with OSA and to make correlations between these parameters and disease severity.

## METHODS

### Study design, ethics and setting

This study was designed as an observational cross-sectional study with a convenience sample. Interim power analyses were performed and detected a 90% statistical power.

The study was conducted in our Departments of Cardiology and Pulmonary Diseases between March 1, 2014, and November 1, 2014. The Ethics Committee of the Afyon Kocatepe University School of Medicine approved this study. All patients and control subjects gave their informed consent prior to inclusion.

### Participants

The study sample included subjects consulted at a sleep laboratory for clinically suspected OSA. These were all the consecutive patients examined over an eight-month period between March 2014 and November 2014. After polysomnographic examination, subjects with five or more obstructive apnea, mixed apnea or hypopnea events per hour were diagnosed as having OSA in accordance with the criteria of the American Academy of Sleep Medicine. Subjects presenting simple snoring, with apnea-hypopnea index less than 5 and no systemic diseases were enrolled in the control group. Subjects with diabetes mellitus, hypertension, ischemic heart disease, heart failure, renal disease, chronic inflammatory diseases, disorders of the autonomic nervous system or endocrine system, histories of drug use affecting the autonomic nervous system, pulmonary diseases or a smoking habit were excluded from the study.

Systolic and diastolic blood pressure of all patients and controls were measured from the right arm using a mercury manometer, after they had rested in a seated position for at least five minutes. Blood pressure was measured using a mechanical sphygmomanometer before polysomnographic evaluation. Patients whose office systolic blood pressure was ≥ 140 mmHg and/or diastolic blood pressure was ≥ 90 mmHg were considered to present arterial hypertension. Body weight and height were also assessed. Body mass index (BMI) was calculated as weight in kilograms divided by the square of height in meters (kg/m^2^).

### HRT and HRV analysis

Full-night Holter monitoring (Reynolds Medical, Pathfinder Software version V8.255) was performed on all subjects. HRT parameters, turbulence onset (TO) and turbulence slope (TS) were calculated using the HRT View software version 0.60-0.1 (downloadable via www.h-r-t.com). All Holter recordings were checked, and any visually observed artifacts that the program accepted as normal premature ventricular beats were excluded from the analyses. TO reflects the initial heart rate acceleration after a premature ventricular beat, whereas TS represents the subsequent heart rate deceleration following a premature ventricular beat. TO was determined as the difference between the mean of the two RR intervals after premature ventricular beats and the mean of the two RR intervals before premature ventricular beats divided by the mean value of two RR intervals before premature ventricular beats. TS was defined as the maximum positive value of the slope of a regression line computed over any sequence of five subsequent sinus RR intervals within the first 15 sinus intervals after premature ventricular beats. TO values determined for all suitable premature ventricular beats were averaged to obtain a final TO value. TO < 0% and TS > 2.5 ms/RR are considered to be normal values.^22^

The HRV parameters used in the present study were selected in accordance with guidelines from the European Society of Cardiology and the North American Society of Pacing and Electrophysiology.^13^ The following time domain HRV parameters were computed: standard deviation of all normal-to-normal (NN) intervals (SDNN), standard deviation of NN interval averages during all five-minute segments across all recordings (SDANN), mean of the standard deviation of all NN intervals for all five-minute segments across all recordings (SDNN index), integral of the density distribution of NN intervals divided by the maximum of the density distribution (HRV triangular index) and root mean square of the sum of squares for differences between adjacent NN intervals (RMSSD).

### Polysomnography

Full-night polysomnography (PSG) was recorded for all subjects using a digital PSG system (E series, Compumedics, Abbotsford, Victoria 3067, Australia) within the Department of Chest Diseases, Afyon Kocatepe University. Respiratory and physical changes were recorded during sleep. For all patients, electroencephalography, electrooculogram and submental electromyography data were obtained. Oronasal airflow was measured using a nasal cannula placed in the nose. Thoracoabdominal movements were evaluated by means of sensors placed on the chest and abdomen to determine respiratory patterns. A pulse oximeter and electrocardiography electrodes were used to measure oxygen saturation and heart rate, respectively. Leg movements were examined using electromyography sensors positioned on the anterior tibialis muscle. Sleep stages were scored in accordance with standard criteria from the American Academia of Sleep Medicine.^23^

Apnea is defined as a drop in the peak signal excursion by ≥ 90% of the pre-event baseline for at least 10 seconds. Hypopnea is an event in which there is a ≥ 30% reduction in nasal pressure signal excursions from baseline and an associated ≥ 4% desaturation from the pre-event baseline for at least 10 seconds. Obstructive events were defined as ongoing thoracoabdominal effort in cases of partial or complete airflow cessation. The apnea-hypopnea index (AHI) was the number of apnea or hypopnea events recorded during the study per hour of sleep. The severity of OSA was defined as mild (AHI ≥ 5 and < 15), moderate (AHI ≥ 15 and ≤ 30) or severe (AHI > 30). The oxygen desaturation index (ODI) was also calculated, as the total number of oxygen desaturation events divided by total duration of sleep.

All polysomnographic data was interpreted by a sleep specialist who was blind to the subjects’ HRT and HRV analysis results.

### Statistical analysis

Statistical analyses were performed using SPSS version 20.0 (IBM Co., Armonk, NY, USA). Data were expressed as the mean ± SD, medians (with interquartile range), or number (and %). Assumptions of normal distribution were tested using the Kolmogorov-Smirnov test. Categorical variables between groups were compared using a chi-square test. Differences between the patient group and control subjects were tested using Student’s t test for parametric variables and the Mann-Whitney U test for nonparametric variables. Comparisons between more than two groups (patients with mild, moderate or severe OSA and control subjects) in relation to variables with homogenous distributions were also analyzed using one-way analysis of variance (ANOVA) and Tukey’s test for post-hoc analyses. Comparisons between more than two groups in which the variables were not normally distributed were made using the Kruskal-Wallis test. In addition, analysis of covariance (ANCOVA) was performed to compare HRT and HRV parameters among the groups, controlling for BMI. Correlations between AHI, TO with AHI, and total oxygen desaturation were analyzed using Spearman’s rho correlation coefficients. An alpha level of P < 0.05 was accepted as significant.

## RESULTS

### Demographic characteristics

The study subjects consisted of 106 newly diagnosed patients with OSA (mean age 49.2 ± 11.2 years) and 27 healthy controls (mean age 47.2 ± 7.9 years). The patients included 30 with mild OSA (mean age 49.1 ± 10.4 years), 34 with moderate OSA (mean age 51.2 ± 6.8 years) and 42 with severe OSA (mean age 54.4 ± 8.3 years) OSA. There were no differences between the groups in terms of age or gender. However, there was a significant difference between the groups in terms of BMI. The subjects’ demographics are depicted in Table 1 and Table 2.

**Table 1.**
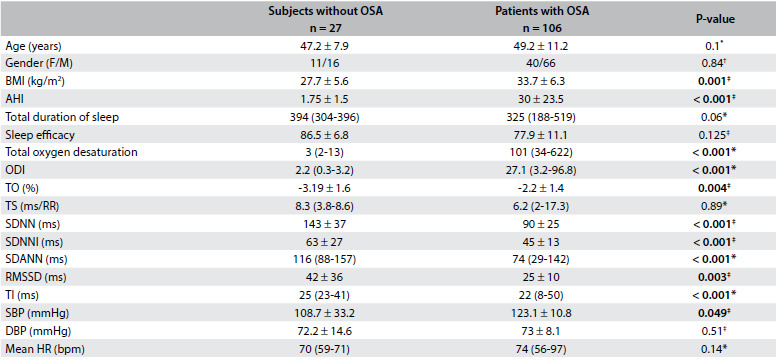
Demographics, polysomnographic results and HRV and HRT parameters among control subjects and patients with OSA

Data were shown as mean ± SD or median (with interquartile range). Categorical variables are defined as percentages. *Mann-Whitney U test; ^†^chi-square test; ^‡^Student’s t test. OSA = obstructive sleep apnea; F = female; M = male; BMI = body mass index; AHI = apnea-hypopnea index; ODI = oxygen desaturation index; TO = turbulence onset; TS = turbulence slope; SDNN = standard deviations of all (normal-to-normal) NN intervals; SDNNI = mean of the standard deviation of all NN intervals for all 5-min segments of the entire recording; SDANN = standard deviation of averages of NN intervals in all 5-min segments of the entire recording; TI = triangular index; RMSSD = the square root of the mean of the sum of the squares of differences between adjacent NN intervals; SBP = systolic blood pressure; DBP = diastolic blood pressure; HR = heart rate; BPM = beats per minute.

**Table 2.**
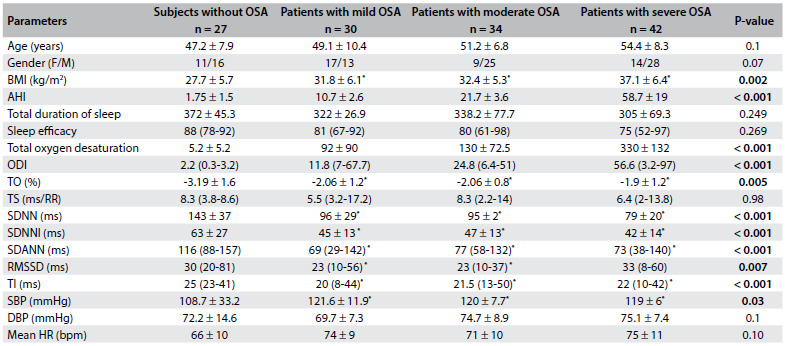
Polysomnographic results and HRT and HRV parameters compared between subgroups

Data are shown as mean ± SD or median (with interquartile range). Categorical variables are defined as percentages. Comparisons between patients with mild, moderate or severe OSA and control subjects were analyzed using the Kruskal-Wallis test or ANOVA and Tukey’s test for post-hoc analyses and significant p values were presented bold. ^*^P < 0.05: between subjects without OSA and patients with mild, moderate and severe OSA. OSA = obstructive sleep apnea; F = female; M = male; BMI: body mass index; AHI = apnea-hypopnea index, ODI = oxygen desaturation index, TO = turbulence onset; TS = turbulence slope; SDNN = standard deviations of all normal-to-normal (NN) intervals; SDNNI = mean of the standard deviation of all NN intervals for all 5-min segments of the entire recording; SDANN = standard deviation of averages of NN intervals in all 5-min segments of the entire recording; TI = triangular index; RMSSD = the square root of the mean of the sum of the squares of differences between adjacent NN intervals; SBP = systolic blood pressure; DBP = diastolic blood pressure; HR = heart rate; BPM = beats per minute.

### HRV and HRT parameters

HRT measurements were obtained from 84 individuals (n = 24 controls, n = 21 with mild OSA, n = 20 with moderate OSA and n = 19 with severe OSA). For the remaining subjects, HRT could not be calculated due to a lack of ventricular premature beats during Holter recording.

No significant differences were found between OSA patients and control subjects in terms of total sleep time, sleep efficacy, age, TS, blood pressure, and heart rate. The TO, SDNN, SDNNI, SDANN, RMSSD, and TI values were significantly lower among OSA patients than among control subjects (Table 1). ANCOVA was performed to compare HRT and HRV parameters between the groups, controlling for BMI. However, significant differences between groups continued after eliminating the influence of BMI.

The subjects were divided into four groups according to their AHI values. The subjects without OSA (n = 27) (AHI < 5), patients with mild OSA (AHI 5-15) (n = 30), patients with moderate OSA (n = 34) and patients with severe OSA (AHI > 30) (n = 42) were compared with regard to HRV, HRT and polysomnographic parameters (Table 2). Post-hoc analyses were performed. No significant differences between mild OSA patients, moderate OSA patients and severe OSA patients in terms of HRT and HRV parameters emerged. However, there were significant differences between mild OSA patients and control subjects, between moderate OSA patients and control subjects and between severe OSA patients and control subjects in terms of TO, SDNN, SDNNI, SDANN, RMSSD and TI values (Table 2).

**Figure 1.**
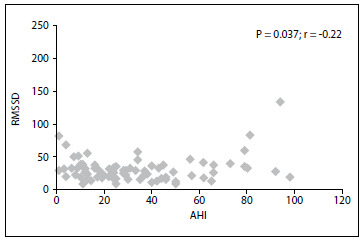
Relationship between apnea hypopnea index (AHI) and root mean square of the successive differences (RMSSD).

When the correlation between AHI (which reflects disease severity) and TO was investigated, no statistically significant correlation was observed (P = 0.356; r = -0.11). There was no statistically significant correlation between total oxygen desaturation and TO (P = 0.631; r = -0.06). There was also no statistically significant correlation between total oxygen desaturation and TO (P = 0.631; r = -0.06). However, RMSSD and AHI were negatively correlated (P = 0.037; r = -0.22; Figure 1).

**Figure 2.**
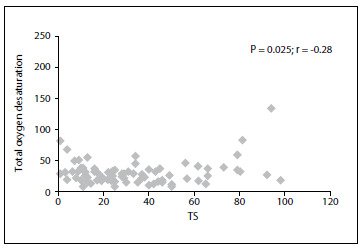
Relationship between total oxygen desaturation and turbulence slope (TS).

 A significant correlation between total oxygen desaturation and TS (P = 0.025; r = -0.28) was observed (Figure 2). In addition, there were negative correlations between ODI and SDNN, SDANN and TI (respectively: P = 0.002, r = -0.28; P < 0.001, r = -0.34; and P = 0.01, r = -0.23).

## DISCUSSION

The findings from the present study revealed that the patients with OSA had lower values for HRV and HRT than those of the control subjects. Such reductions were observed even in patients with mild OSA. Although there was no significant difference between patient groups in terms of HRV and HRT parameters, significant correlations between TS and total oxygen desaturation, between RMSSD and AHI, and between ODI and SDNN, SDANN and TI were found.

Previous studies revealed that HRV and HRT worsen in ­OSA.^19^^,^^20^^,^^21^^,^^24^^,^^25^^,^^26^^,^^27^ The authors of these studies thought that this deterioration might be due to hypoxia during periods of apnea. Ari et al.^19^ reported that HRT worsened in OSA while HRV did not change and there was a relationship between HRT and AHI. Aytemir et al.^20^ observed increased myocardial vulnerability and autonomic nervous system imbalance in OSA cases. They found that HRT, HRV and QT dynamicity parameters were significantly worse among patients with OSA. Furthermore, a correlation was revealed between AHI and HRT. However, they did not find any relationship between AHI and HRV. These authors^20^ also showed the presence of autonomic balance changes in favor of the sympathetic nervous system at night and hypoxemia relating to apnea. Erdem et al.^25^ investigated HRT parameters among patients with pure OSA. They found that the OSA group had a significantly higher mean TO than the control group, and that the AHI of the OSA group was positively correlated with TO. Our study supports the findings of previous studies. TO was significantly higher in patients with OSA than in the control group and, although there were no correlations between AHI and HRT parameters, there was a correlation between total oxygen desaturation and TS in our study.

Yang et al.^21^ also showed that HRV did not change while HRT worsened during sleep among patients with OSA, and that alterations in nighttime HRT correlated with sleep-disordered breathing severity. This indicates the existence of abnormalities in autonomic cardiac activity within moderate-to-severe OSA, even in the absence of evident cardiac disease. In contrast, we did not determine any significant differences between the patient groups (mild, moderate and severe OSA) in terms of HRT and HRT parameters. These differences in results between the present study and the study by Yang et al.^21^ might be attributable to differences in study group selection. Yang et al.^21^ grouped their patients as mild and moderate to severe, while we grouped our patients as mild, moderate and severe. In addition, merely one overnight polysomnographic assessment might not provide enough information regarding the severity of OSA. The duration of OSA might also be a factor affecting the deterioration of autonomic cardiac function.

D’Addio et al.^27^ investigated the effects of pathological respiratory patterns on HRT among patients with severe OSA. They found that TS increased during apnea but observed decreases during normal intervals following an apnea event (here, OSA patients showed a higher sympathetic tone). This supports the idea that autonomic cardiac function is impaired due to hypoxia during apnea.

Unlike Yang^21^ and Ari et al.,^19^ Lado et al.^28^ found that all HRV parameters decreased during sleep among patients with moderate and severe OSA, compared with a normal, healthy group. In addition, among patients with severe low-frequency band and high-frequency band OSA indices, the total HRV power was lower during intervals labeled as apneas than those labeled as normal. In the present study, although the lowest values for HRV parameters in OSA patients compared with controls were determined; this decrease did not reach statistically significance level among the patient groups. However, we found correlations between some HRV parameters and the values of ODI and AHI. Therefore, these correlations constitute the strength of our research.

The relatively small sample size was a limitation for the present study. Moreover, we only evaluated night-time measurements on HRV and HRT. Thus, further research evaluating both day and night time values is needed in order to contribute important and new data to the literature, given that these parameters are affected by the breathing pattern of OSA patients during sleep. The arrhythmia mechanism in OSA is closely related to apnea and hyperventilation events, which depend on the sympathovagal balance.^29^ There is dominance of parasympathetic tone and decreased sympathetic activity during sleep among healthy subjects.^30^ However, this situation can change in OSA. Parasympathetic activity, with slowing of the heart rate, is dominant during periods of apnea. When subsequent apnea termination and temporary arousal from sleep occur, sympathetic activity predominates with resultant heart rate acceleration.^31^ Therefore, increased sympathetic tone and baroreflex dysfunction can cause cardiac arrhythmia and sudden death. Sympathetic tone can be evaluated through provocative testing and spectral HRV analysis. The lack of power of the spectral analysis of heart rate and absence of provocative testing are other limitations of the present study. Lastly, HRT parameters could not be calculated in 21% of the patients, who did not present a premature ventricular beat.

## CONCLUSION

HRT and HRV parameters were different in OSA patients than in control subjects. Correlations could be made between the severity of apnea that could be determined through AHI, ODI and total oxygen desaturation, and such parameters seemed to be present. These subjects should be followed up in order to monitor any further adverse outcomes.
